# Healthy Habits, Physical Activity, Stress, and Food Consumption Trends in Chilean Adults with Irritable Bowel Syndrome during the COVID-19 Pandemic

**DOI:** 10.3390/ijerph21050533

**Published:** 2024-04-25

**Authors:** Carolina Mandiola-Palma, Camila Leiva, María Jesús Moya-Salazar, Eliane A. Goicochea-Palomino, Hans Contreras-Pulache, Jeel Moya-Salazar

**Affiliations:** 1 Faculties of Health Science, Universidad Autónoma de Chile, Santiago 83201, Chile; caromanpal@gmail.com (C.M.-P.); camila.leiva.tamayo@hotmail.com (C.L.); 2 Qualitative Unite, Nesh Hubbs, Lima 51001, Peru; majums2005@gmail.com; 3 School of Medical Technologist, Faculties of Health Science, Universidad Tecnologica del Perú, Lima 51001, Peru; elagoichi@gmail.com; 4 Digital Transformation Center, Universidad Norbert Wiener, Lima 51001, Peru; hans.contreras@uwiener.edu.pe; 5 Faculties of Health Science, Universidad Privada del Norte, Lima 51001, Peru

**Keywords:** irritable bowel syndrome, lifestyles, COVID-19, stress, diet, alcohol, Chile

## Abstract

The COVID-19 pandemic has increased stress levels in the population due to radical lifestyle changes caused by containment measures. Studies suggest that high levels of stress may be related to the development of irritable bowel syndrome (IBS). This study aims to explain how quarantine habits and lifestyles acted as risk factors for the frequency of this syndrome during the COVID-19 pandemic. An observational study was conducted with 34 Chilean participants (average age 24.5 ± 3.85 years), of whom 21 (62%) were female. Surveys on consumption trends and lifestyles created by the authors were administered. Additionally, we used the global physical activity questionnaire (GPAQ) and the depression anxiety stress scales (DASS-21) to assess psychological stress and the Rome IV criteria to assess IBS. Significant differences were found between individuals with better healthy habits compared to those with poor healthy habits. The former showed lower sedentary activity (32%, *p* = 0.005), only 27% were fast eaters (vs. 44%, *p* = 0.001), had shorter nap intervals (14% vs. 28%, *p* = 0.03), and higher vegetable consumption (*p* = 0.02). There were 20 cases (59%) of IBS, with a strong association with the female sex (*p* = 0.004), where females were 15 times more likely to develop it compared to males (*p* = 0.008). Additionally, when alcohol consumption was added to females, there was a higher likelihood of developing this syndrome (*p* = 0.009), as individuals who consumed alcohol were 12 times more likely to develop it compared to those who did not (*p* = 0.02). Among other factors, it was observed that 57% of those with the syndrome consumed drinks more often (*p* = 0.02) but consumed fewer nuts (*p* = 0.009). In conclusion, IBS has a multifactorial etiology, and correcting individual habits such as alcohol consumption could potentially prevent or delay its development. Therefore, it is important to maintain healthy lifestyles, regardless of non-modifiable factors such as gender, in order to better cope with this syndrome.

## 1. Introduction

Irritable bowel syndrome (IBS) is defined as a multifactorial gastrointestinal disease that produces a range of disruptive symptoms in daily life, including abdominal pain and discomfort, bloating, and alterations in stool consistency and frequency [1,2]. The root of these symptoms lies in disruptions in intestinal motility and sensitivity. Intense intestinal contractions often trigger diarrhea or constipation, depending on their speed or slowness of occurrence [3]. Regarding its etiology, the exact cause of IBS remains largely uncertain. Experts such as Blomhoff, Spetalen, Jacobsen, and Malt have suggested that stress may be a triggering factor in the development of the pathophysiology of this disease [1]. On the other hand, some researchers link the etiology of IBS to environmental factors, infections, intestinal inflammation, and alterations in gut flora [4].

Amid the COVID-19 pandemic, both Chile and the global community confronted a pivotal public health emergency that irreversibly altered the social landscape. By mid-2023, the total number of COVID-19 cases in Chile reached 1,590,887, with a total of 34,016 deaths [5]. Epidemic outbreaks such as this profoundly impact the behavior and daily routines of the population, often resulting in psychological distress encompassing anxiety, depression, and stress. In this scenario, a noticeable surge in stress levels was evident in the population, with more than 70% experiencing mild or elevated stress levels. This surge was primarily linked to ongoing concerns regarding the risk of SARS-CoV-2 infection and profound lifestyle changes enforced by containment measures [6]. These measures, including national isolation and social distancing (such as nighttime curfews and the closure of educational and government institutions) [7], as well as international measures (such as border closures) [5], generated ongoing tension in society. Disruption of social interactions and the radical transformation of daily routines exacerbated emotional and mental tensions in the population.

Stress significantly influences various aspects of the digestive system, including permeability, mobility, visceral sensitivity, blood flow, and secretions. This impact is notably associated with irritable bowel syndrome (IBS), which is intricately connected to the brain–gut–microbiota axis [8]. IBS is influenced by stress and exhibits a global prevalence of 10–20%, with a notable prevalence of 26.2% in Chile. The constipation-predominant IBS (IBS-C) subtype is the most common, affecting women more frequently in a 2:1 ratio compared to men [2,9]. Considering that confinement can lead to increased stress levels in individuals, which in turn can lead to various health problems, one of which is the impact on the gastrointestinal system, it is relevant to explore the relationship between stress and IBS.

Therefore, this study aims to assess the increase in the development of this disease in the context of the COVID-19 lockdown, analyzing factors such as diet, physical activity, healthy habits, and stress levels as possible contributors to IBS.

As previously reported, COVID-19 has significantly altered the trajectory of various illnesses, including cancer [10]. Consequently, our study posits the following hypotheses: (i) the pandemic has induced changes in dietary patterns during the lockdown, (ii) there are gender-based disparities in IBS cases, with a higher impact on women, (iii) alcohol consumption plays a significant role in IBS development, and (iv) specific dietary trends during lockdown might serve as indicators for IBS patients, such as reduced fiber and nut intake. Evaluating these aspects is crucial given the diverse dietary and social shifts during the pandemic, especially considering the differences among countries. In Latin America, with its heterogeneous populations, understanding IBS behavior becomes essential.

## 2. Methods

### 2.1. Study Design and Settings

This is an observational study. The onset of the pandemic in Chile began with the confirmation of the first case on 3 March 2020. From this point onward, there was a rapid increase in the number of cases, leading to the implementation of key prevention measures such as national isolation and social distancing. These measures included the closure of schools, universities, municipalities, and districts, as well as the implementation of night-time curfews [7]. Internationally, land, sea, and air border closures were also enforced, restricting the movement of foreign individuals, a measure that remained in effect until July 2021 [5].

### 2.2. Population and Inclusion Criteria

**Figure 1:**
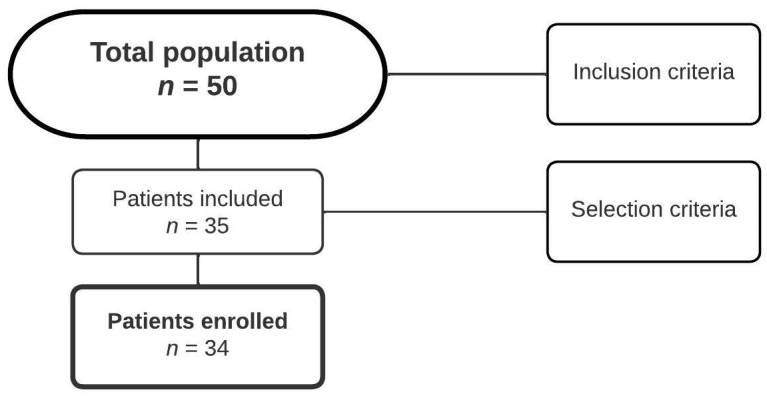
Flowchart of participant selection in the study.

We employed a non-probabilistic convenience sampling method, selecting individuals available on the Universidad Autónoma de Chile’s contact list. The response rate among potential adult participants was 40%, and we included the 50 respondents who agreed to participate. All participants were adults residing in Santiago, Chile. Participants were recruited voluntarily through email and online platforms (i.e., Instagram), utilizing the contact networks of the university database (Figure 1).

Inclusion criteria were adults of both genders, without any disease indicating an inflammatory process (i.e., systemic lupus erythematosus), and a willingness to engage in physical activity. We excluded individuals under pharmacotherapy related to a gastrointestinal disease, patients diagnosed with depression and receiving pharmacological treatment for it, individuals diagnosed with IBS, individuals with pre-existing gastrointestinal disorders, and patients affected by non-communicable chronic diseases (i.e., diabetes or hypertension). Before participating in the study, the subjects provided informed consent through a pre-study signature before completing the surveys.

### 2.3. Instruments

The research was conducted through a clinical assessment of IBS using the criteria established by Rome IV [11]. These criteria allow the characterization, classification, and categorization of functional gastrointestinal disease. For the diagnosis of IBS, abdominal pain is considered in conjunction with changes in stool form, enabling its characterization into different categories. In addition to clinical evaluation, various surveys were administered to estimate the lifestyles, dietary trends, physical activity [12], and psychological stress [13] of the participants, as detailed below.

A dietary trend survey was implemented with the purpose of investigating the dietary habits of the respondents and verifying if they could influence the development of IBS. The survey analyzed the intake and frequency of energy, protein, carbohydrate, and lipid consumption, with a specific focus on saturated and monounsaturated fatty acids. Food groups were quantified and included cereals, fruits, vegetables, meat, eggs, legumes, dairy products, fats and oils, sugars and sweeteners, and alcohol and tobacco (Supplementary Table S1). This survey provided essential information regarding the dietary habits of the respondents, allowing the identification of possible relationships between inadequate nutrient intake and IBS. The survey was quantified using the chemical composition table of Chilean foods [14].

We employed a lifestyle survey specifically designed for this study and validated by the research committee of the Universidad Autónoma de Chile (Supplementary Table S2). This survey facilitated the identification of whether individuals had incorporated healthy lifestyle habits into their daily routines. Comprising 26 Likert-like items, the survey delved into various aspects, including the number of meals consumed, their frequency (ranging from “Never”, “Once a week 2–4 times a week”, “5–6 times a week”, and “Every day”), and details regarding the quantity and temperature of the foods and drinks ingested. Additionally, the Global Physical Activity Questionnaire (GPAQ) [12] was included, which allowed us to assess whether individuals engaged in physical activity and, if so, how an increase in it could contribute to the improvement of IBS-related symptoms. Each individual was classified based on the amount of daily physical activity, both work-related and recreational, thus categorizing them as sedentary, moderately active, or highly active.

Finally, recognizing the significant role of stress in the development of IBS, the DASS-21 Psychological Stress Survey [13] was employed. This survey consists of 21 questions on a Likert-type scale that assesses levels of depression, anxiety, and stress. By classifying responses into categories such as “never”, “sometimes”, “often”, “frequently”, and “always”, we were able to determine the individual’s position on the scale of these psychological dimensions. We considered DASS-21 stress scale scores (items: 1, 6, 8, 11, 12, 14, and 18) of 13 to 16 points (severe stress) and 17 or more (extreme severe stress) as high stress [15]. The questionnaire was distributed in Spanish and previously validated in the Chilean population [16]. This analysis was essential for identifying possible triggering factors of symptoms related to IBS.

### 2.4. Interviews, Data Collection, and Analysis

All interviews were intended to administer survey instruments. Following initial contact through social media and phone calls, interviews were conducted using the GoogleMeet platform, and surveys were digitally administered through GoogleForms^TM^ (both from Google, Sunnyvale, CA, USA). These approximately 45-minute interviews were structured to assess participants’ habits and lifestyles, specifically focusing on the frequency of IBS during the COVID-19 lockdown. Subsequently, a second interview was arranged five days later to gather insights into consumption trends [17]. This follow-up phase occurred through video calls or phone calls, aiming to investigate the potential impact of dietary habits adopted during quarantine on the increased frequency of IBS.

The data were collected directly from digital forms and nutritional data collection sheets. These data were processed and coded using SPSS v24.0 (IBM, Armonk, NY, USA), where statistical analyses were conducted. Initially, descriptive analysis was performed to calculate simple frequencies, means, and standard deviations. Subsequently, Student’s *t*-tests and chi-square tests were applied to identify differences in lifestyles and dietary habits between two groups: those with more outstanding healthy habits (scoring 4–5 points) compared to the group with less healthy habits (scoring 0–3 points), as well as between patients with IBS and a healthy control group (*n* = 34). Finally, a linear regression analysis was conducted, considering a significance threshold of *p* < 0.05 for all the tests performed.

### 2.5. Ethical Aspects

This study was approved by the Institutional Review Board (IRB) of the Universidad Autónoma de Chile (UAC-2020-115-2020-12-121) and complied with the guidelines of the Declaration of Helsinki [18].

## 3. Results

Fifty participants were initially identified, but only 34 (68%) met the study’s inclusion criteria. The average age of the 34 participants was 24.5 ± 3.85 years (range 20 to 40 years), and 21 (62%) were women. Each participant belonged to the middle-class demographic and had successfully completed their secondary education. All participants worked in a home office at least 5 days a week. While there were no significant differences in terms of weight, age, body mass index (BMI), smoking habits, and stress levels between groups with good and poor healthy habits, a highly significant difference was noted in relation to the level of physical activity. There was a clear reduction in the percentage of sedentary activity (32%, *p* = 0.005) among individuals who maintained healthier habits (Table 1).

Regarding dietary patterns, notable differences were revealed in eating habits, with 27% of individuals adopting healthier habits compared to 44% of those with less healthy habits (*p* = 0.001). Differences were also observed in the habit of taking naps, with 14% in the group with better healthy habits compared to 28% in the less healthy habits group (*p* = 0.03). Lastly, a significant difference was noted in the vegetable intake category (*p* = 0.02). In the rest of the analyzed factors, no significant differences were found (Table 2).

IBS was present in 20 (59%) of the surveyed individuals, of whom 45% were female and had a high level of stress. When analyzing the lifestyles of these participants, significant differences were observed in certain factors, such as gender (*p* = 0.004), as it was evident that female individuals were more predisposed to the development of this gastrointestinal disease. Other factors included drink consumption (*p* = 0.02) and especially alcohol consumption (*p* = 0.009), with a higher likelihood of developing IBS in individuals who consumed alcohol compared to those who did not. Additionally, participants with IBS consumed fewer nuts than healthy controls (23.9 ± 36.4 vs. 93.9 ± 89.3 g, *p* = 0.009). The rest of the evaluated factors did not show a significant difference related to this syndrome (Table 3).

When analyzing all factors that had significant differences in the presence of IBS, gender and alcohol were found to be the most influential (Table 4). In the case of gender, females were 15 times more likely to develop this syndrome compared to males (*p* = 0.008). When evaluating the alcohol factor, individuals who consumed alcohol were found to have 12 times higher chances of developing this syndrome compared to individuals who did not consume alcohol (*p* = 0.02).

## 4. Discussion

This study demonstrated that more than half of the 34 participants presented with symptoms compatible with IBS. Notably, this prevalence was predominantly present in women (particularly those with a high level of stress) and those subjects who consumed alcohol. In terms of consumption habits, it was observed that individuals with IBS consumed drinks more often, while their intake of nuts was significantly reduced. Furthermore, statistically significant differences were highlighted between individuals who maintained healthy lifestyles compared to those who did not. Specifically, those with healthy habits were less prone to sedentary activity and had a slower pattern of food consumption in terms of speed, shorter nap intervals, and increased vegetable consumption.

### 4.1. Strengths and Limitations

This study, to the best of the authors’ knowledge, is the first research into the interaction between quarantine-induced habits, lifestyle choices, and risk factors contributing to the development of irritable bowel syndrome (IBS) in the Chilean population during the COVID-19 pandemic. Some symptoms of COVID-19 infection in patients with IBS have previously been described [19], but comprehensive evaluations of their dietary and lifestyle factors have not been developed. The study had a comprehensive scope, covering multifaceted aspects such as lifestyle patterns, food consumption frequency, physical activity, and psychological stress [11,12,13,14,15]. In addition, its distinctive contribution lies in addressing the scarcity of global studies quantifying IBS cases during the pandemic [20,21,22,23,24], making it a noteworthy scientific advancement, particularly in the Latin American context [25].

This study identified certain limitations. First, considering the Rome IV criteria, their levels of specificity and sensitivity are acknowledged to be suboptimal. Certain questions within these criteria, crucial for diagnosing IBS, may be considered complex or difficult to comprehend, particularly among rural or economically disadvantaged populations [26]. Particularly, the study omitted the inclusion of additional factors such as environmental agents, infections, or alterations in the intestinal flora, which also contribute to the etiology of IBS [4]. A further limitation of the study is the small sample size. It is essential to extrapolate the findings of this research by including larger cohort studies.

### 4.2. Main Findings and Clinical Implications

IBS has emerged as the most prevalent functional gastrointestinal condition globally, with an incidence of 10–20% [8], reaching 15% in Latin America [27] and a significant 26.2% in the population of the Metropolitan Area of Chile. This syndrome predominantly affects women, with a 2:1 ratio compared to men [3,4]. The study’s results support this trend, indicating that IBS development is primarily observed in women, granting them up to 15 times more likelihood of experiencing it than men. This gender disparity is rooted in hormonal factors, as the interaction between gut bacteria and the brain occurs through the gut–brain microbiome axis, a system potentially influenced by sex hormones, particularly estrogen [28].

Alcohol consumption is another notable factor influencing IBS development. The analysis reveals that individuals who consume alcohol have a 12-fold higher risk of developing the syndrome compared to those who abstain from alcohol consumption (*p* = 0.02). This correlation is explained by alcohol’s impact on intestinal absorption and permeability, as well as its ability to alter gastrointestinal motility. This effect is generated through a neuronal mechanism characterized by the reduction in immunoreactive neurons in the jejunum, particularly in individuals with a chronic history of alcohol consumption [29]. This phenomenon is directly related to a decrease in intestinal transit time, especially when consuming excessive alcohol, which can precipitate or intensify symptoms such as abdominal pain, nausea, and indigestion [30]. However, no similar pattern was observed in other habits, such as tobacco and coffee consumption. Particularly, evidence suggests that caffeine tends to exacerbate symptoms, not only due to its ability to increase gastric acid secretion but also because of its effect on the motor activity of the rectosigmoid and colon [29]. Further studies are required to understand the degree of alcohol and other dietary factors’ influence during COVID-19 restrictions.

As previously mentioned, IBS stands out as the most predominant functional gastrointestinal disease, manifesting as a response to stress [30]. This response has been characterized by the activation of the sympathetic nervous system and the hypothalamic–pituitary–adrenal axis in various contexts [8,31]. In fact, scientific literature has shown that over 50% of IBS patients link the onset of their symptoms to episodes of high stress [32]. Considering the increase in stress in the population due to the COVID-19 pandemic and, in particular, contingency measures such as quarantine [6], it would be reasonable to infer a relationship between this factor and the development or exacerbation of IBS symptoms. However, our findings did not show significant differences based on their dietary habits.

In our population, 59% were IBS cases; in Japan, in an even larger sample, the prevalence was 16.6% (857/5157), and 11.9% of respondents reported worsening gastrointestinal symptoms, although it was also not directly related to stress, anxiety, or depression [22]. On the other hand, in Bulgaria, both the prevalence of IBS and gastrointestinal symptoms were higher during the pandemic (26.3% vs. 20.0% pre-pandemic; 68.9% vs. 56.0% pre-pandemic, respectively), and anxiety was associated with the presence and/or increase in all gastrointestinal symptoms [23]. These differences may be due to population, epidemiological, and social differences, such as the level of stress related to COVID-19 cases and death peaks, control measures such as quarantine and social distancing, different lifestyles, cultural factors, and dietary habits, which should be explored in multicenter studies at each stage of the pandemic.

On the other hand, the available information supported by experts suggests that these patients do not face a significantly higher risk of SARS-CoV-2 infection or developing COVID-19. However, according to the international SECURE-IBD registry [21], patients with severe inflammatory bowel disease who also contract COVID-19 have a higher probability of requiring hospitalization. This hospitalization may be related to underlying intestinal disease and COVID-19 infection or possibly a combination of both conditions [20]. Studies in the context of Latin America yielded similar results, with a mortality rate of 1.7% and 7.4% of cases experiencing a combination of factors such as admission to the Intensive Care Unit, need for respiratory assistance, and/or death. Furthermore, the use of specific medications to treat inflammatory bowel diseases was not associated with a worsening of COVID-19 status [25]. It is relevant to note that a meta-analysis revealed that patients who recover from COVID-19 have an increased risk of developing IBS, regardless of their country of origin, including North America, Europe, and the Middle East/Asia [24].

As we know, the COVID-19 pandemic had a significant impact on mental health, leading to disorders such as anxiety, stress, and depression [32], which have been associated with changes in dietary habits [33]. Specifically, there was an increase in emotional eating due to higher depressive and anxiety symptoms, while binge eating occurred due to increased stress [34]. Despite this information, various studies show positive trends towards the consumption of healthy foods and reduced intake of foods with lower nutritional value [35]. For example, in Spain and Chile, there was increased consumption of fruits (27% and 54%, respectively), legumes (22.5% and 72%, respectively), and vegetables (21% and 67%, respectively), as well as a reduction in the consumption of processed meats (35.5%)—in Chile, hot dogs (90%) and processed meats (73%)—and sugary drinks (32.8% and 79%, respectively) [36,37]. However, countries like Argentina and Peru experienced negative changes, indicating consumption of less healthy and inadequate foods (44.7% and 58.8%, respectively) during the pandemic [20,38].

In response to these findings, medical professionals provide information and emphasize the importance of self-care through dietary guidelines that can contribute favorably to the reduction in IBS symptoms. This can be achieved by excluding foods that promote the development of this syndrome, such as the low-FODMAP diet, which restricts certain fermentable carbohydrates [39]. These compounds can pass through the colon without being absorbed and produce IBS symptoms; however, following the diet 100% is necessary to prevent this from happening [40,41,42]. According to scientific evidence, it is recommended to exclude foods containing lactose. Lactose intolerance is more intense in those who suffer from the syndrome because they are hypersensitive, and it can worsen the symptoms of the syndrome [29]. Finally, there are few studies that demonstrate whether a gluten-free exclusion diet contributes to the deterioration or improvement of IBS signs and symptoms [42].

## 5. Conclusions

This research offers a detailed insight into the risk factors and lifestyle habits related to IBS in the Chilean population. While significant differences were identified in certain aspects, such as gender and alcohol consumption, further research is required to fully understand the complex interactions contributing to the development of this gastrointestinal disease. These findings can be valuable in guiding prevention and treatment strategies and promoting healthy lifestyle habits among those at risk of developing IBS.

## Figures and Tables

**Table 1:** General characteristics of the studied population.

Characteristics	Healthy Habits		*p*-Value
	Bad Habits (*n* = 16)	Good Habits (*n* = 18)	
Age (years)	24.9 ± 4.5	24.2 ± 3.2	0.48
Weight (kg)	73.04 ± 15.9	67.9 ± 16.0	0.43
BMI (kg/mt^2^)	26.3 ± 5.4	24.6 ± 3.5	0.27
Female (%)	73	50	0.47
Smokers (%)	13	36	0.15
Sedentary activity (%) **	41	32	0.005 *
High stress levels (%) ***	34	77	0.07

* *p* < 0.05 (significant). ** Obtained with the GPAQ. *** Obtained with the DASS-21.

**Table 2:** Dietary habits of participants during the COVID-19 pandemic. * *p* < 0.05 (significant).

Characteristics	Variable	Healthy Habits		*p*-Value
		Bad Habits (*n* = 16)	Good Habits (*n* = 18)	
Lifestyle	Irregular pattern (%)	3	0	0.07
	Fast eater (%)	44	27	0.001 *
	Drink consumption (%)	25	18	0.15
	Nap interval (%)	28	14	0.03 *
	Fat consumption (%)	6	5	0.07
Dietary intake	Energy (Kcal)	2048.6 ± 498.4	1889.2 ± 446.04	0.32
	Proteins (g)	73.06 ± 19.9	76.4 ± 21.3	0.75
	Lipids (g)	81.7 ± 22.7	69.9 ± 21.9	0.10
	Saturated Fatty Acids (g)	24.02 ± 10.8	19.9 ± 10.5	0.09
	Monounsaturated Fatty Acids (g)	28.7 ± 11.7	25.2 ± 8.5	0.32
	Carbohydrates (g)	235.8 ± 86.9	229.3 ± 64.8	0.84
	Fiber (g)	32.6 ± 18.8	30.7 ± 9.6	0.82
Dietary intake by food group	Vegetables (g)	42.9 ± 19.1	69.0 ± 39.8	0.02 *
	Fruits (g)	116.5 ± 79.01	130.2 ± 100.4	0.41
	Red meat (g)	52.3 ± 44.8	53.1 ± 63.5	0.73
	White meat (g)	52.9 ± 40.6	57.8 ± 55.1	0.82
	Legumes (g)	54.4 ± 33.8	79.7 ± 64.5	0.21
	Nuts (g)	76.7 ± 80.01	55.3 ± 79.9	0.52
	Caffeine (g)	50 ± 154.9	22.2 ± 94.2	0.49
	Sweets (g)	194.2 ± 120.6	193.2 ± 248.4	0.59

**Table 3:** Lifestyles of patients with IBS during the COVID-19 pandemic. * *p* < 0.05 (significant).

Characteristics	Variables	IBS Cases (*n* = 20)	Controls (*n* = 14)	*p*-Value
Clinical and dietary	Female (%)	92.8	45	0.004 *
	Age (years)	25.2 ± 4.8	24.1 ± 3.0	0.44
	BMI (Kg/mt^2^)	26.9 ± 5.3	24.4 ± 3.6	0.11
Lifestyles	Irregular pattern (%)	0	5	0.39
	Fast eaters (%)	64	55	0.59
	Drink consumption (%)	57	20	0.02
	Nap intervals (%)	29	40	0.49
	Fat consumption (%)	7	10	0.77
	Smoking (%)	29	40	0.49
	Alcohol (%)	50	7	0.009 *
	Sedentary activity (%)	64	55	0.58
	High stress level (%)	86	80	0.66
Dietary intake	Energy (Kcal)	1994.7 ± 574.1	1943 ± 398.4	0.76
	Proteins (g)	74.3 ± 20.03	75.1 ± 21.2	0.91
	Lipids (g)	7.7 ± 25.02	73.5 ± 21.9	0.61
	Saturated Fatty Acids (g)	23.3 ± 10.5	20.4 ± 10.07	0.41
	Monounsaturated Fatty Acids (g)	27.03 ± 11.5	26.7 ± 9.4	0.94
	Carbohydrates (g)	234.7 ± 72.6	230.8 ± 78.3	0.88
	Fiber (g)	33.9 ± 19.6	29.9 ± 9.7	0.44
Dietary intake by food group	Vegetables (g)	59.0 ± 30.3	59.4 ± 36.3	0.64
	Fruits (g)	100.6 ± 49.4	139.9 ± 108.1	0.21
	Red meat (g)	52.7 ± 52.8	52.7 ± 57.3	0.99
	White meat (g)	52.2 ± 45.3	57.8 ± 51.1	0.74
	Legumes (g)	71.1 ± 69.08	65.4 ± 40.4	0.76
	Nuts (g)	23.9 ± 36.4	93.9 ± 89.3	0.009 *
	Caffeine (g)	42.8 ± 160.3	30 ± 97.8	0.77
	Sweets (g)	192.4 ± 223.6	207.1 ± 176.8	0.83

**Table 4:** Models of analyses of dietary habits in IBS patients.

IBS	Dietary Habits		*p*-Value
	Bad Habits	Good Habits	
Crude model	1	0.61 (0.15–2.43)	0.48
Model 1	1	15.4 (1.67–142.30)	0.008
Model 2	1	12.58 (1.35–116.98)	0.02

Model 1: gender. Model 2: alcohol.

## Data Availability

The data presented in this study are available on request from the corresponding author.

## Supplementary Materials

The following supporting information can be downloaded at: https://www.mdpi.com/article/10.3390/ijerph21050533/s1, Table S1: Quantified Survey on Food Consumption Trends; Table S2: Lifestyle assessment.
