# Supplementary material for: Healthy Habits, Physical Activity, Stress, and Food Consumption Trends in Chilean Adults with Irritable Bowel Syndrome during the COVID-19 Pandemic
Source: Int J Environ Res Public Health. 2024 Apr 25;21(5):533. doi: 10.3390/ijerph21050533 (PMC11121439; doi:10.3390/ijerph21050533)
Supplement: Supplementary file 1 [file ijerph-21-00533-s001.zip › ijerph-2670675-supplementary.pdf]

CODE

Suppl.1. Quantified Survey on Food Consumption Trends

Name:

Date:

INSTRUCTIONS

**1.- Cereals**	**Never or hardly ever**	**Every month**	**Every week**			**Every day**			
		1-3	1	2-4	5-6	1	2-3	4-6	+6
Whole wheat bread (2 slices)									
Marraqueta bread (1/2 unit)									
Hallulla bread (1/2 unit)									
White sandwich bread (2 slices)									
Rice (3/4 cup)									
Noodles (1 cup)									
Oats (4 tablespoons)									
Breakfast cereals (check the portion on the package)									
Boiled potatoes (egg-sized unit)									
Others: please specify									

For each food item, mark the box that indicates your usual consumption frequency. Each food item will have a specified portion, and if you consume more than the portion, please specify how much.

**2.- Vegetables**	**Never or**	**Ever**	**Every week**	**Every day**

	**hardly ever**	**y month**							
		1-3	1	2-4	5-6	1	2-3	4-6	+6
Swiss chard and cooked spinach (1/2 cup)									
Cauliflower, broccoli, zucchini (1 cup)									
Tomato (regular-sized)									
Cooked carrot (1/2 cup)									
Green beans (1 cup)									
Butternut squash (1/2 cup)									
Mushrooms (1/2 cup)									
Cooked beetroot (1/2 cup)									
Lettuce, endive, cabbage, celery, cucumber (1 cup)									
Others:									

**3.- Fruits**<br>**Describe the fruits you consume regularly and in what portions.**	**Never or hardly ever**	**Every month**	**Every week**			**Every day**			
		1-3	1	2-4	5-6	1	2-3	4-6	+6
Apple (regular-sized)									
Orange (regular-sized)									
Pear (regular-sized)									
Banana (half unit or one small banana)									
Plum (2 small units)									
Pineapple (1 slice 20 x 1 cm)									

Strawberries (1 cup)									
Grapes (10 to 12 units)									
Others:									

**4.- Meats**<br>**Describe the meats you regularly consume and in what portions.**<br>**Describe processed meats: Ham (turkey, chicken, or pork), other cured meats, and their portions (6 x 6 x 1 cm).**	**Never or hardly ever**	**Every month**	**Every week**			**Every day**			
		1-3	1	2-4	5-6	1	2-3	4-6	+6
Chicken									
Red meat									
Fish									

**5.- Eggs and legumes**	**Never or hardl**	**Every**	**Every week**	**Every day**

	y ever	m on th							
		1-3	1	2-4	5-6	1	2-3	4-6	+6
Whole egg (1 UNIT)									
Egg white									
Egg yolk									
Describe the legumes you consume, the frequency, and in what portion (beans, lentils, chickpeas, split peas, soy) (3/4 cup).									
Beans									
Lentils									
Split peas									
Chickpeas									

**6.- Dairy**	**Never or hardly ever**	**Every month**	**Every week**			**Every day**			
		1-3	1	2-4	5-6	1	2-3	4-6	+6
Skim milk (200 mL or 1 cup)									
Semi-skimmed milk (200 mL or 1 cup)									
Whole milk (200 mL or 1 cup)									
Flavored milk (200 mL or 1 cup)									
Cultured milk (200 mL or 1 cup)									
Skimmed milk powder (heaping tablespoon or 3 level teaspoons)									

Semi-skimmed milk powder (heaping tablespoon or 3 level teaspoons)									
Whole milk powder (heaping tablespoon or 3 level teaspoons)									
Full-fat yogurt (120 g)									
Skim yogurt (120 g)									
Cheese (1 slice)									
Cottage cheese (1 slice, 3 cm thick)									
Cream cheese									
Other dairy: please specify									

**5.- Fats and oils**	**Never or hardly ever**	**Every month**	**Every week**			**Every day**			
		1-3	1	2-4	5-6	1	2-3	4-6	+6
Sunflower oil (1 teaspoon)									
Olive oil (1 teaspoon)									
Other oil (1 teaspoon)									
Butter (1 teaspoon)									
Margarine (1 teaspoon)									
Cream (1 teaspoon)									
Peanuts (25 units)									
Walnuts (3 units)									
Almonds (15 units)									
Avocado (3 tablespoons or half a small avocado)									
Pâté	NO								

Otros: Especifique									

**6.- Sugars**	**Never or hardly ever**	**Every month**	**Every week**			**Every day**			
		1-3	1	2-4	5-6	1	2-3	4-6	+6
Sugar	NO								
Honey		3					2		
Palm honey	NO								
Sweets/candies				3		1			
Ice cream		1							
Sweet cookies				3		1			
Sugary beverages	NO								
Sugary juices	NO								
Cakes and pastries		1							
Others. Please, specify									

**7.- Sweeteners**	**Never or hardly ever**	**Every month**	**Every week**			**Every day**			
		1-3	1	2-4	5-6	1	2-3	4-6	+6
Stevia	NO								
Stevia/sucralose	NO								
Sucralose	NO								
Saccharin	NO								

Aspartame	NO								
Light drinks	NO								
Zero drinks	NO								
Other products with sweeteners, please specify									

**8.- Alcohol and tobacco consumption: Please specify the type of alcohol you typically consume and the quantities in which you consume it.**	**Never or hardly ever**	**Every month**	**Every week**			**Every day**			
Alcohols:	NO	1-3	1	2-4	5-6	1	2-3	4-6	+6
Tobacco:	NO								
Cigarrettes	NO								
Tabacco	NO								
If you have ever smoked, please indicate how long it has been since you quit smoking									

CODE

Suppl. 2. LIFESTYLE ASSESSMENT

1.- MEALS FREQUENCY	1	2	+3
How many meals do you have during the day, including snacks?			
How many meals did you have **before the quarantine**?			

2.- Regularidad de las comidas **durante la cuarentena**	Never	Once a week	2-4 times a week	5-6 times a week	Every day
Breakfast					
Lunch					
Afternoon snack					
Dinner					
Portions					

2.1- Frequency of meals **before quarantine**	Never	Once a week	2-4 times a week	5-6 times a week	Every day
Breakfast					
Lunch					
Afternoon snack					
Dinner					
Portions					

3.- Drink consumption during meals **during quarantine**	Never	Sometimes	Frequently	Always

Drink consumption before meals				
Drink consumption during meals				
Drink consumption after meals				

3.1- Drink consumption during meals **<u>before the quarantine</u>**	Never	Sometimes	Frequently	Always
Drink consumption before meals				
Drink consumption during meals				
Drink consumption after meals				

4.- Amount of drinks consumed during meals **<u>during quarantine.</u>**	≤1 cup	2-3 cups	3-4 cups	+4 cups
4.1- Amount of drinks consumed **<u>during</u>** meals **<u>before quarantine.</u>**	≤1 cup	2-3 cups	3-4 cups	+4 cups
5.- Temperature of beverages **during quarantine**	Room temperature	Cold	Very cold	
5.- Temperature of beverages **before quarantine**	Room temperature	Cold	Very cold	

6.- Food intake				
6.1.- How well did you chew your food during meals **during quarantine?**	Not very well	Well	Very well	
6.1.- How well did you chew your food during meals **before quarantine?**	Not very well	Well	Very well	
6.2.- How much time did you take for lunch or dinner **during quarantine?**	I never have lunch or dinner.	<10 min	10-20 min	>20 min
6.2.- How much time did you take for lunch or dinner **before quarantine?**	I never have lunch or dinner.	<10 min	10-20 min	>20 min
6.3 How soon after eating did you have a snack **during quarantine?**	I never take a nap.	<30 min	0.5 a 2 hours	> 4 hours
6.4 How soon after eating did you have a snack **before quarantine?**	I never have a snack.	<30 min	0.5 a 2 hours	> 2 hourse
